# Loss of the obscurin-RhoGEF downregulates RhoA signaling and increases microtentacle formation and attachment of breast epithelial cells

**DOI:** 10.18632/oncotarget.2338

**Published:** 2014-08-10

**Authors:** Nicole A. Perry, Michele I. Vitolo, Stuart S. Martin, Aikaterini Kontrogianni-Konstantopoulos

**Affiliations:** ^1^ Department of Biochemistry and Molecular Biology, University of Maryland School of Medicine, Baltimore, MD; ^2^ Department of Physiology, University of Maryland School of Medicine, Baltimore, MD; ^3^ Marlene and Stewart Greenebaum National Cancer Institute Cancer Center, University of Maryland School of Medicine, Baltimore, MD

**Keywords:** Breast cancer, metastasis, obscurin, actomyosin contractility, paclitaxel, circulating tumor cells, microtentacles

## Abstract

Obscurins are RhoGEF-containing proteins whose downregulation has been implicated in the development and progression of breast cancer. Herein, we aim to elucidate the mechanism for increased motility of obscurin-deficient cells. We show that shRNA-mediated obscurin downregulation in MCF10A cells leads to >50% reduction in RhoA activity relative to scramble control (shCtrl), as well as decreased phosphorylation of RhoA effectors, including myosin light chain phosphatase, myosin light chain, lim kinase, and cofilin, in both attached and suspended cells. These alterations result in decreased actomyosin contractility, allowing suspended cells to escape detachment-induced apoptosis. Moreover, ~40% of shObsc-expressing cells, but only ~10% of shCtrl-expressing cells, extend microtentacles, tubulin-based projections that mediate the attachment of circulating tumor cells to endothelium. Indeed, we show that MCF10A cells expressing shObsc attach *in vitro* more readily than shCtrl cells, an advantage that persists following taxane exposure. Overall, our data suggest that loss of obscurins may represent a substantial selective advantage for breast epithelial cells during metastasis, and that treatment with paclitaxel may exacerbate this advantage by preferentially allowing obscurin-deficient, stem-like cells to attach to the endothelium of distant sites, a first step towards colonizing metastatic tumors.

## INTRODUCTION

Neoplastic transformation of epithelial cells is both caused by and results in inappropriate changes to a variety of cell signaling pathways [1-3]. In particular, deregulation of actin and microtubule cytoskeletons results in increased invasive potential of many tumor types, allowing transformed cells to migrate away from the primary tumor, survive within the vasculature, and reattach to and extravasate through the endothelium to colonize distant sites [4-6]. The actin cytoskeleton is regulated largely through the dynamic activation and inactivation of Rho-family GTPases, which, through modification of their effector proteins, control such processes as actomyosin contractility, cellular polarity, and cell spreading [7]. While the consequences of altered Rho GTPase signaling have been shown extensively in transformed cells cultured on a rigid substrate [8-11], effects on suspended cells, such as those found within the vasculature as circulating tumor cells (CTCs), are only now being elucidated. Recent studies have revealed that modifications which result in increased actin filament turnover, such as activation of the actin-severing protein cofilin, cause a weakening of the actin cortex of suspended cells [12, 13]; this imbalance in cytoskeletal forces permits the development of tubulin-based projections termed “microtentacles” (McTNs) [14, 15]. McTN formation by breast epithelial cells is associated with increased metastatic potential and endothelial attachment [14, 16, 17], particularly following treatment with the microtubule stabilizing drug, paclitaxel (Taxol) [18, 19].

In light of the discovery that the human *OBSCN* gene is highly mutated in a number of solid tumors [20, 21], we demonstrated that giant obscurins, once thought to be expressed exclusively in striated muscles, are abundantly expressed in normal breast, skin, and colon cell lines, and breast tissue, but nearly absent from cancer cells and tumors [22]. Abrogation of giant obscurins from non-tumorigenic MCF10A breast epithelial cells using shRNA technology (shObsc) resulted in increased apoptotic resistance following etoposide treatment [22], as well as increased migration, invasion, and both primary and metastatic tumor formation in mice [23]. While these investigations highlight a number of phenotypic changes, including increased actin dynamics and the induction of an epithelial-to-mesenchymal transition (EMT), no direct link between the loss of obscurins and cytoskeletal alterations has been demonstrated yet. The aim of the present study is to characterize changes to Rho GTPase signaling induced upon loss of obscurins, as occurs during the progression of breast cancer. We found that decreased activation of RhoA and its effectors in obscurin-deficient MCF10A cells causes decreased susceptibility to detachment-dependent apoptosis and increased microtentacle (McTN) formation. These functional differences persist following paclitaxel treatment, after which the obscurin shRNA-treated cells attach more efficiently than scramble control (shCtrl) cells. This data suggests that the decrease in RhoA signaling in obscurin-deficient cells is responsible, at least in part, for their ability to survive more robustly at multiple steps of the metastatic cascade [23], culminating in their enhanced ability to colonize distant sites, even in the presence of Taxol.

## RESULTS AND DISCUSSION

**Figure 1:**
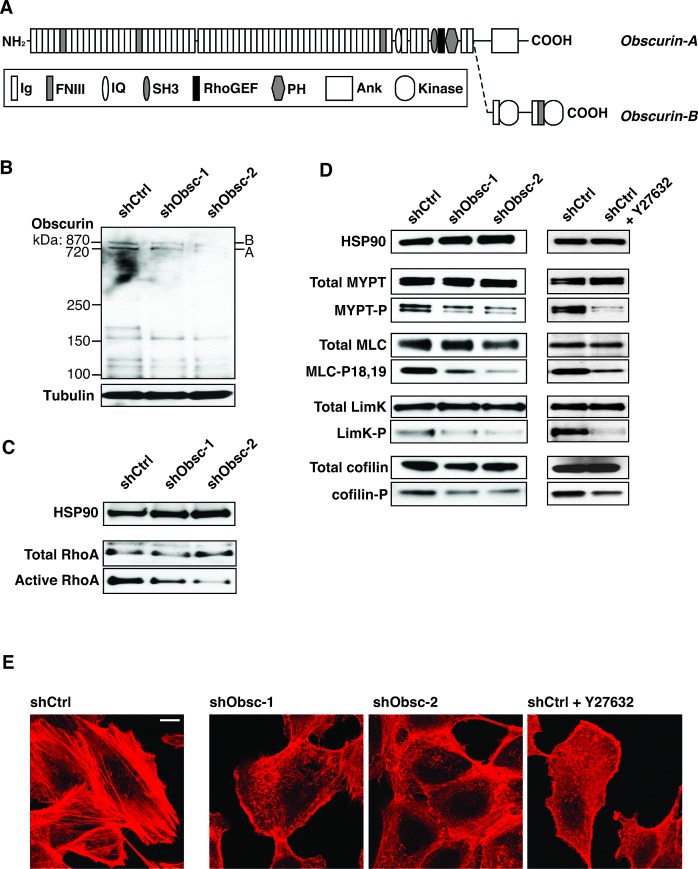
Loss of obscurins downregulates RhoA signaling in attached MCF10A cells A) Schematic of the giant isoforms of obscurin A and B. Domain types: Ig: Immunoglobulin; FNIII: Fibronectin type-III; IQ: Isoleucine-Glutamine calmodulin-binding motif; SH3: Src-Homology-3; RhoGEF: Rho Guanine Exchange Factor; PH: Pleckstrin Homology; Ank: Ankyrin binding motif; Kinase: Serine/Threonine Kinase. B) Expression of giant isoforms A and B, is significantly downregulated in MCF10A cells stably transduced with obscurin shRNA-1 or-2, targeting Ig24 and Ig9, respectively, but not scramble control (shCtrl). Tubulin serves as loading control. C) Activation of RhoA is decreased in attached MCF10A cells expressing either shObsc-1 or-2. HSP90 and total RhoA serve as loading controls. D) Phosphorylation of RhoA effectors in attached cells is decreased when shObsc-1 or -2 is expressed, similar to treatment of shCtrl cells with 10 μM of the ROCK inhibitor Y27632. HSP90 serves as loading control. E) MCF10A cells expressing shObsc-1 or -2 or treated with Y27632 form fewer stress fibers compared to control cells. The scale bar (upper right in shCtrl panel) indicates 10 μM.

Obscurins, originally characterized in skeletal and cardiac muscle, play key roles in the development of the sarcomere and its contractile function [24-32]. They are encoded by the single *OBSCN* gene, located on human chromosome 1q42, and produced via alternative splicing [31, 33]. The largest isoforms, obscurin-A (720 kDa) and obscurin-B (870 kDa), are comprised of immunoglobulin and fibronectin-III repeats followed by several signaling and scaffolding domains, including an isoleucine-glutamine calmodulin binding domain, a src-homology-3 (SH3) domain, and tandem Rho guanine exchange factor (RhoGEF) – pleckstrin homology (PH) motifs. The giant isoforms differ only in their extreme C-termini; while obscurin-A has a non-modular C-terminus that includes binding sites for ankyrins as well as predicted phosphorylation sites for ERK kinases [32, 33], obscurin-B includes two active serine-threonine kinases in its C-terminus [31, 33, 34] (Figure 1A). Recent evidence suggests that multiple alternatively spliced smaller isoforms exist as well [34, 35].

Recent observations from our group revealed the presence of increased mobile actin in obscurin shRNA-treated MCF10A cells [23], which led us to hypothesize that obscurins may play a role in the regulation of the actin cytoskeleton. Moreover, it has been previously shown that ectopic expression of the obscurin RhoGEF motif in COS-7 cells or mouse tibialis anterior muscle results in increased GTP-bound RhoA [36]. Therefore, we hypothesized that in MCF10A breast epithelial cells, knockdown of giant obscurins would result in decreased RhoA activity and a concomitant reduction of RhoA-driven processes downstream of the Rho Activated Kinase (ROCK). Indeed, in MCF10A cells that stably express shRNA to obscurins (shObsc-1 or shObsc-2, collectively referred to as “shObsc”; Figure 1B, densitometry in Figure S1A), RhoA activity is reduced to levels approximately half of control when grown in a monolayer (Figure 1C; densitometry in Figure S1B). As a consequence, we also observe a dramatic (>50%) decrease in the phosphorylation of ROCK targets, including myosin light chain phosphatase (MYPT), myosin light chain (MLC), and lim kinase (LimK), as well as the LimK target cofilin (Figure 1D; densitometry in Figure S1C). Importantly, the effect of obscurin knockdown on target phosphorylation is very similar to the effect of ROCK inhibition by Y27632 (Figure 1D), consistent with it acting in the RhoA-ROCK pathway.

The shObsc-mediated effect of reduced activating phosphorylation of MYPT, a phosphatase for MLC, along with decreased direct ROCK-mediated activation of MLC, is clearly demonstrated by morphological changes to the actin cytoskeleton. Whereas shCtrl-expressing cells readily assemble stress fibers due to persistent RhoA-driven phosphorylation of MLC, shObsc cells have fewer stress fibers and instead exhibit short, disorganized actin filaments, similar to the effect of ROCK inhibition by Y27632 (Figure 1E). The altered actin morphology indicates that depletion of obscurins causes attenuation of MLC-induced actomyosin contractility; this loss of contractility and the associated loss of adhesion [37] are likely at least partially responsible for the enhanced migratory characteristics observed in shObsc MCF10A cells [11, 23, 38].

**Figure 2:**
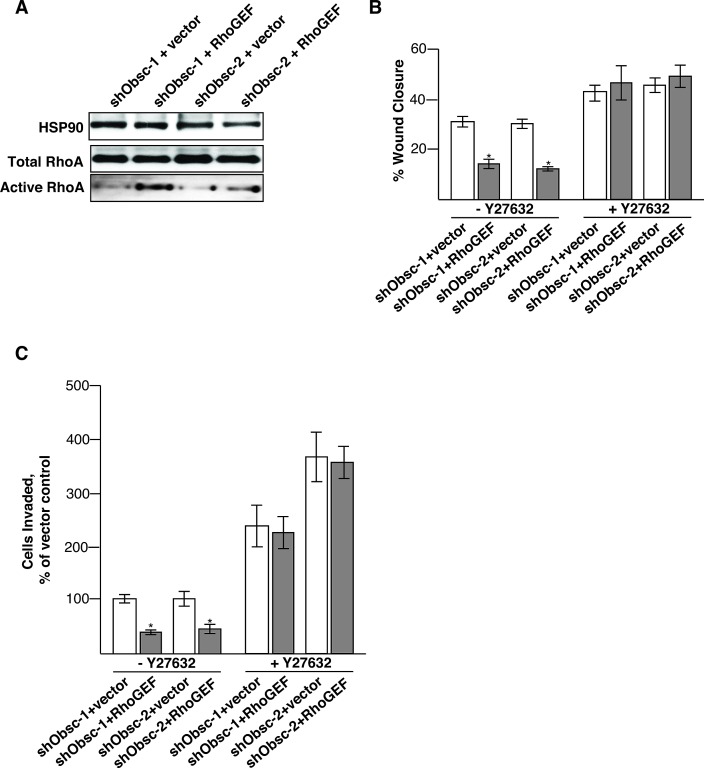
Transient expression of the obscurin RhoGEF domain decreases migration and invasion in obscurin-deficient MCF10A cells A) Transient expression of the obscurin RhoGEF domain, but not vector control, is sufficient to re-activate RhoA in obscurin knockdown cells, as shown by immunoblot analysis of total and active RhoA. B) After 6 hours, shObsc-1 and -2 MCF10A cells expressing the obscurin RhoGEF domain have closed a monolayer wound approximately 50% less than those expressing vector control. Y27632 treatment increases the rate of wound closure for shObsc-transfected cells without a statistically significant difference between ectopic expression of vector control or RhoGEF. N=3. Error Bars: +/- S.D. Asterisks: *p*<0.05. C) After 16 hours, 60% fewer shObsc-1 and -2 cells expressing the obscurin RhoGEF domain have invaded through matrigel towards a chemoattractant than those expressing vector control. Y27632 treatment increases the rate of invasion for shObsc-transfected cells without a statistically significant difference between ectopic expression of vector control or RhoGEF. N=3. Error Bars: +/- S.D. Asterisks: *p*<0.05.

Although the effects we saw on RhoA effector phosphorylation and stress fiber formation upon loss of obscurins are consistent with our hypothesis that loss of the obscurin RhoGEF motif reduces RhoA activity in breast epithelial cells, we wanted to confirm that this is not an indirect consequence of treatment of MCF10A cells with shRNA. To this end, we transfected shObsc MCF10A cells with a cDNA encoding the human obscurin RhoGEF domain, or vector control. Because of the strong dominant negative effect previously observed following overexpression of the RhoGEF domain in cells with intact endogenous large obscurins [36], we elected to not perform the experiments in shCtrl MCF10A cells, which still express high levels of the large isoforms (Figure 1B). Transient transfection with the obscurin RhoGEF domain rescues the shObsc-induced decrease in RhoA activity (Figure 2A), demonstrating that the changes to RhoA signaling observed in breast epithelial cells upon obscurin downregulation are specifically due to the loss of the RhoGEF activity. Furthermore, we extended our findings from our previous work [23], which showed increased migration and invasion upon loss of giant obscurins, by demonstrating that ectopic expression of the obscurin RhoGEF in obscurin knockdown MCF10A cells reduces both monolayer migration (Figure 2B) and invasion through matrigel (Figure 2C). Treatment with the ROCK inhibitor Y27632 reversed the suppressive effects of the RhoGEF domain for both monolayer migration (Figure 2B) and invasion (Figure 2C), as shown by the absence of statistically significant differences between vector control- and RhoGEF-expressing obscurin knockdown cells following Y27632 treatment, indicating that the obscurin RhoGEF acts upstream of ROCK by modifying RhoA activity. Interestingly though, Y27632 treatment modestly increased migration and substantially increased invasion of the obscurin knockdown MCF10A cells. This is consistent with previous findings obtained from a variety of cell types for both migration [39, 40] and invasion [41, 42], and likely reflects the fact that complete ROCK inhibition blocks signal inputs from several cellular pathways, while knocking down obscurins has a more modest effect due to its specificity, affecting only RhoA signaling to ROCK.

The observation of reduced phosphorylation (i.e., increased activity) of cofilin suggests that in addition to the reduced actomyosin contractility, obscurin knockdown enhances actin-severing activity and turnover of actin filaments, consistent with increased mobile actin detected in shObsc-expressing MCF10A cells [23]. Previous measurements in PTEN^-/-^ MCF10A cells have demonstrated that higher cofilin activity results in decreased cortical actin tension [12]. This decrease in tension is no longer sufficient to counteract the extension of microtubules, which in suspended epithelial cells (such as those that are found within the vasculature as circulating tumor cells, CTCs) allows the formation of microtentacles, McTNs [14, 15], tubulin-based projections that allow reattachment to the endothelium. We therefore focused the remainder of our studies on suspended cells and how the depletion of obscurins may govern processes related to metastasis.

**Figure 3:**
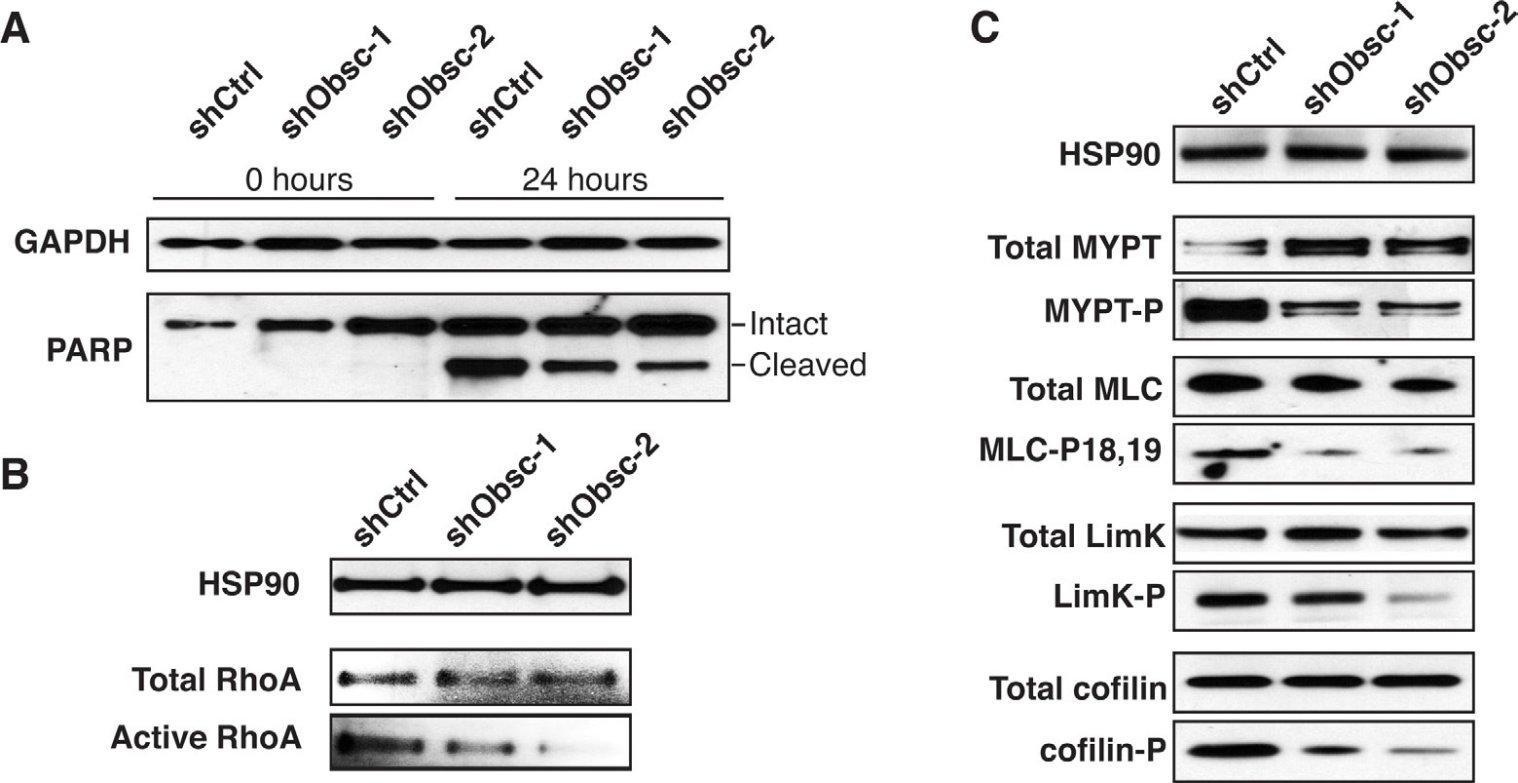
Loss of obscurins decreases apoptosis and RhoA signaling in suspended MCF10A cells A) Decreased PARP cleavage in shObsc-1 and -2 cells indicates reduced apoptosis after 24 hours in suspension. B) Activation of RhoA is decreased in suspended MCF10A cells expressing shObsc-1 or -2. HSP90 and total RhoA serve as loading controls. C) Phosphorylation of RhoA effectors in suspended cells is decreased when shObsc-1 or -2 is expressed. HSP90 serves as loading control.

Since MCF10A cells expressing shObsc are capable of resisting apoptosis induced by DNA damage [22], we wanted first to see if the apoptotic resistance extended to substrate detachment as well. Epithelial cells rely on survival signals from engaged integrins; in the signals' absence, epithelial cells undergo anoikis [43-45]. Following plating in conditions that prohibit attachment, shCtrl-expressing cells readily undergo programmed cell death, as indicated by cleavage of the caspase-3 substrate, poly-ADP-ribose-polymerase (PARP) (Figure 3A). In contrast, though, shObsc MCF10A cells cleave less PARP after 24 hours, an effect that is abrogated by the addition of the ROCK inhibitor Y27632, which acts downstream of the obscurin RhoGEF's activation of RhoA (Figure S2A). This finding suggests that upon loss of obscurins expression, epithelial cells lose the ability to self-destruct when detached from the extracellular matrix (ECM); this represents an important positive selection step for survival as a CTC and eventual metastatic colonization. The RhoA pathway has previously been implicated in the regulation of apoptosis through actomyosin contractility. MDA-MB-231 breast cancer cells can be re-sensitized to anoikis by overexpression of tropomyosin-1, which activates ROCK-mediated processes such as stress fiber formation [46], and is consistent with several studies demonstrating that ROCK inhibition results in increased survival [47-51]. Furthermore, activation of RhoA by the RhoGEF Abr plays a role in initiating actomyosin contractility and dissociation-induced apoptosis in human embryonic stem cells [52]. Therefore, loss of obscurin RhoGEF-driven RhoA activation is likely responsible for the resistance to detachment-induced apoptosis in shObsc MCF10A cells. Indeed, reduction of RhoA activity (Figure 3B; densitometry in Figure S2B) and effector phosphorylation (Figure 3C; densitometry in Figure S2C) persist even in suspended conditions in obscurin shRNA-expressing MCF10A cells relative to shCtrl.

**Figure 4:**
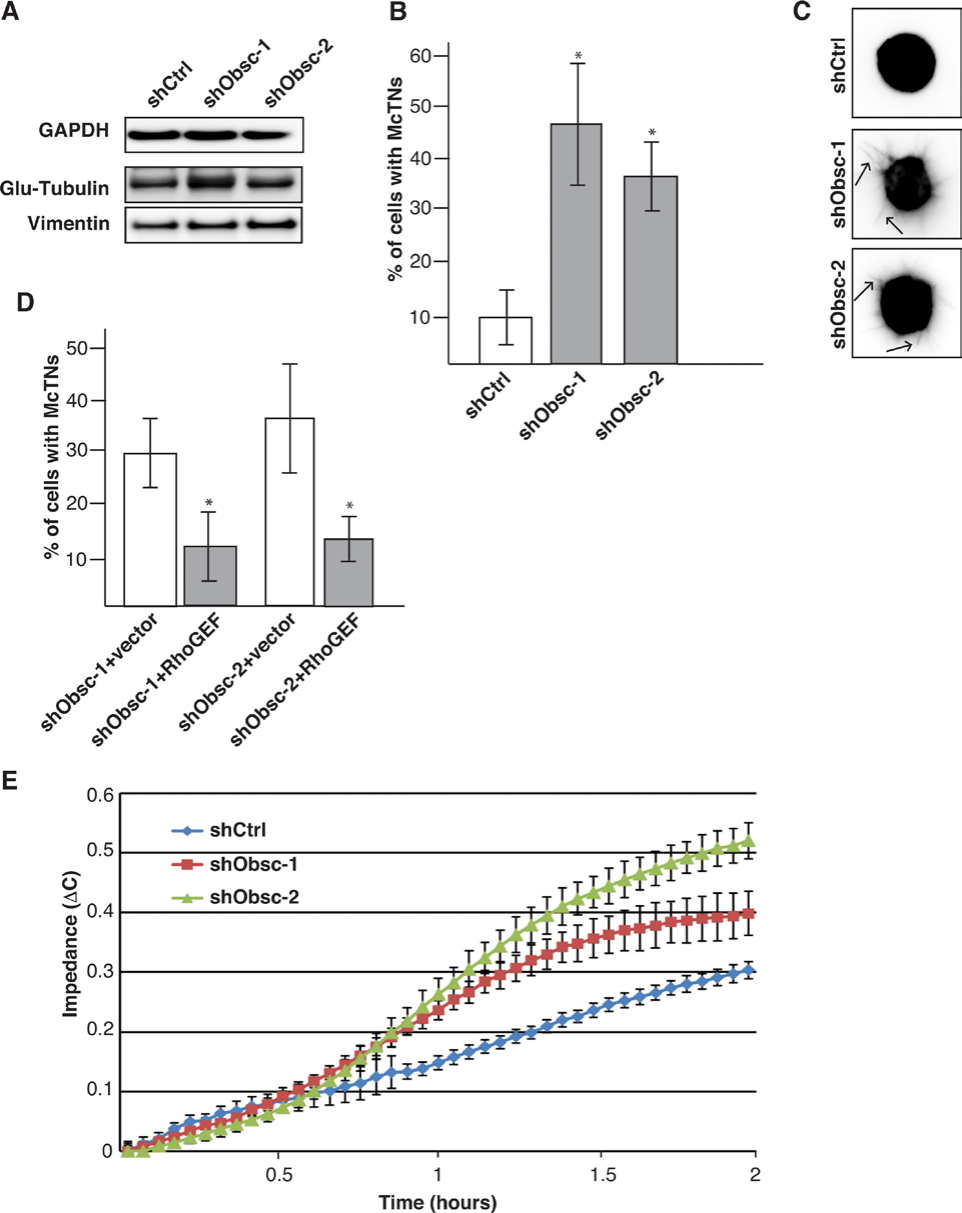
Obscurin knockdown in MCF10A cells causes increased microtentacle formation and attachment A) MCF10A obscurin knockdown cells express more detyrosinated tubulin (glu-tubulin) and vimentin than shCtrl cells, as shown by immunoblot analysis. GAPDH serves as loading control. B) Approximately 40% of MCF10A cells expressing shObsc-1 or -2 form microtentacles compared to 10% shCtrl cells. N=3. Error Bars: +/- S.D. Asterisks: *p*<0.05. C) Representative images of suspended cells expressing shCtrl, shObsc-1 or -2. Arrows in the shObsc panels indicate McTNs. D) The obscurin RhoGEF domain, but not mCherry vector control, is sufficient to abrogate McTN formation in obscurin knockdown cells. N=3. Error Bars: +/- S.D. Asterisks: *p*<0.05. E) An impedance assay indicates that MCF10A cells expressing shObsc-1 or -2 attach more readily than cells expressing shCtrl. Data shown is representative of three independent replicates.

Having ascertained that obscurin knockdown cells have acquired the capacity to survive in detached conditions, we then examined their ability to form McTNs. McTN formation is enhanced when actin is rapidly turning over [12], as is the case in shObsc cells in suspension, as indicated by the decreased cofilin phosphorylation relative to shCtrl cells (Figure 3C). Furthermore, modest concomitant upregulation of detyrosinated α-tubulin (glu-tubulin) and of the intermediate filament vimentin (Figure 4A, densitometry in Figure S3), both of which serve to stabilize microtubules, provides shObsc-expressing cells an additional mechanism to enhance the establishment of McTNs [14, 15]. As a result, while only 10% of shCtrl cells extend robust McTNs in suspension, approximately 40% of shObsc-expressing cells that form McTNs (Figure 4B). This phenomenon is clearly seen in Figure 4C, which depicts representative cells in suspension following staining with the plasma membrane marker CellMask Orange. While shCtrl cells are compact and do not readily form projections, shObsc-expressing cells extend multiple McTNs per cell (arrows). Importantly, McTN formation is disrupted by transient transfection of the obscurin RhoGEF but not vector control, demonstrating that loss of obscurin and the associated downregulation of RhoA-driven processes is sufficient for the extension of microtentacles in MCF10A cells (Figure 4D).

In breast cancer cell lines, the percentage of cells exhibiting McTNs correlates with their *in vitro* metastatic potential [14]. Furthermore, the ability of injected breast tumor cells to reattach in the lungs of mice is associated with the percentage of cells that form McTNs [16]. We thus evaluated the functionality of the McTNs produced by the shObsc-expressing MCF10A cells using an impedance assay of cell reattachment. As shown in Figure 4E, McTN-producing shObsc cells attach approximately 1.25-1.5 fold better than shCtrl cells between one and two hours. However, in the presence of colchicine (a microtubule polymerization inhibitor), attachment is entirely abolished, consistent with a microtubule-driven attachment process (data not shown). Therefore, we conclude that in the absence of giant obscurins, altered actin dynamics allowed the formation of microtentacles, which substantially enhances the ability of obscurin knockdown cells to attach. We have previously observed increased metastasis of shObsc cells relative to shCtrl cells in both subcutaneous and tail-vein mouse tumor models [23]. It is likely that the increased attachment to an xCelligence plate is recapitulated *in vivo* and is partially responsible for the increased metastatic colonization by shObsc cells. Short-term capillary retention experiments [16] could address this question by evaluating the *in vivo* attachment of shCtrl and shObsc cells. Furthermore, testing the ability of the RhoGEF domain to affect reattachment of shObsc cells *in vitro* and *in vivo* will demonstrate its role in abrogating reattachment, and thus metastasis.

**Figure 5:**
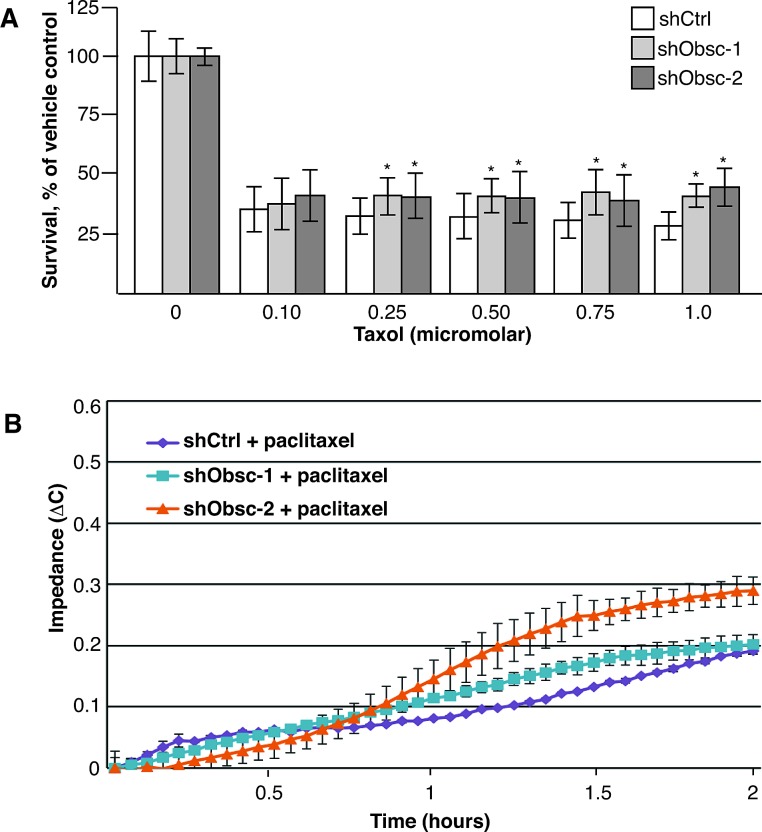
Loss of obscurins allows increased survival and attachment following paclitaxel treatment A) shObsc cells have a survival advantage over shCtrl cells following 24 hour treatment with 0.1-1.0 μM paclitaxel in an XTT assay. N=3. Error Bars: +/- S.D. Asterisks: *p*<0.05. B) An impedance assay indicates that MCF10A cells expressing shObsc-1 or -2 attach more readily than cells expressing shCtrl even in the presence of 0.5 μg/mL paclitaxel. Data shown is representative of three independent replicates.

Given the effect of obscurin knockdown on the formation of microtubule-based projections, we hypothesized that treatment with paclitaxel, which binds to β-tubulin and inhibits disassembly of microtubules, would differentially affect shCtrl- and shObsc-expressing cells. We first treated adherent, proliferating cells with physiologically relevant concentrations of paclitaxel, ranging from 100-1000 nM, and noted a small, but reproducible, difference in viability following a 48-hour exposure (Figure 5A). Importantly, during treatment with 0.5 μg/mL (439 nM) paclitaxel, shObsc cells still exhibited increased capacity to attach to a substrate, albeit at levels lower than untreated cells (Figure 5B). It is known that paclitaxel can cause more than a 1,000-fold increase in the number of CTCs shed from the primary tumor [53]. Given that cells that do not express giant obscurins are relatively resistant to detachment-induced apoptosis, they will survive in the anchorage independent environment of the vasculature. Therefore, not only do low obscurin-expressing or obscurin-depleted tumor cells have a survival advantage, they also have an enhanced ability to form McTNs and attach to distant sites even in the presence of paclitaxel. Thus, tumor cells with low or depleted obscurin expression possess advantages at multiple steps in the metastatic cascade, which could each contribute to their increased metastatic capacity [23].

## CONCLUSIONS

**Figure 6:**
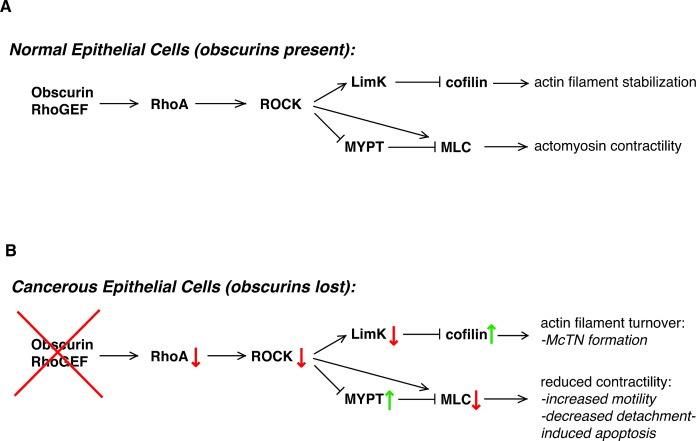
Loss of obscurins alters RhoA-driven signaling and cytoskeletal processes in breast epithelial cells A) In normal cells, the obscurin RhoGEF domain activates RhoA signaling, which then activates ROCK to phosphorylate its downstream effectors. This includes phosphorylation of LimK, which in turn phosphorylates and downregulates the activity of cofilin, resulting in relatively low basal actin turnover. Furthermore, MLC activity (and therefore actomyosin contractility) is relatively high owing to both direct ROCK phosphorylation of MLC as well as inactivating phosphorylation of MYPT, which then dephosphorylates and inactivates MLC. B) In cancer cells that have lost expression of giant obscurins, there is less GTP-bound RhoA (red arrow) owing to less activation by the absent obscurin RhoGEF domain (red X). This causes decreased ROCK activity (red arrow) and less LimK phosphorylation (red arrow); reduced phosphorylation of cofilin by LimK causes an increase in its actin-severing activity (green arrow) and greater actin filament turnover. Reduced MLC activity (red arrow) and thus actomyosin contractility is due to both decreased ROCK phosphorylation as well as increased phosphatase activity by MYPT (green arrow).

The process of metastasis involves many discrete steps at which selective pressure is applied to those cells that escape the primary tumor. A metastatic tumor contains cells that have proliferated at the primary site, invaded away from the primary tumor and intravasated, survived their journey through the vasculature, reattached to distant endothelial cells and extravasated, then commenced proliferating again [54]. Gene expression differences that result in a small positive selection bias at each step will ultimately cause the enrichment of highly resistant cells. In this study along with our previous work, we have shown that loss of expression of obscurins, as is observed in breast cancers [23, 55], is sufficient to establish this selection bias: Cells deficient in obscurins 1) display enhanced stem-like characteristics and proliferate to form tumorspheres [23], 2) migrate and invade through the extracellular matrix [23], 3) survive in anchorage-independent conditions [22], 4) reattach to a substrate (this study), and 5) grow into metastatic tumors [23]. In part, these differences are due to the reduced phosphorylation of the RhoA effectors LimK, cofilin, MYPT, and MLC, causing the increased migration, invasion, and reattachment of obscurin-deficient cells (Figure 6). Importantly, treatment with paclitaxel gives shObsc cells a further survival advantage; these surviving paclitaxel-treated shObsc cells may then reattach more readily than paclitaxel-treated control cells. Therefore, paclitaxel treatment of breast cancer patients could preferentially select for the obscurin-deficient, attachment-competent circulating tumor cells while having an overall cytotoxic effect. Although detailed studies of the effect of paclitaxel on breast cancer cells which have lost expression of obscurins are necessary to truly evaluate risk, it is clear that loss of obscurins constitutes a substantial risk factor for metastatic tumor formation.

## MATERIALS AND METHODS

### Tissue Culture

MCF10A cells stably expressing shCtrl or shObsc-1 or -2 (targeting Ig domains 24 and 9, respectively) were prepared and maintained as previously described [22, 23]. Cells were kept at low passage, never allowed to become confluent, and regularly checked by western blot for knockdown of obscurins.

### Western Blotting

For attached cell blotting, MCF10A cells expressing shCtrl or shObsc were grown to 50% confluence, and fresh complete media was added for 24 hours before lysates were collected in Radio-Immuno-Precipitation Assay (RIPA) or Lammelli buffer. Equal amounts of lysates were electrophoresed and blotted as previously described [12, 22]. For suspended cell blotting, shCtrl or shObsc MCF10A cells were trypsinized and suspended in low-attachment conditions in complete media for 1 hour, before collection for lysate preparation. For ROCK inhibition, 10 μM Y27632 (Sigma) was added to the cells in fresh complete media for 1 hour prior to collection. Antibodies used included obscurin-COOH [56], α-tubulin (Sigma), MYPT and phospho-MYPT (Millipore), MLC and phospho-MLC (Cell Signaling Technology, CST), LimK (Abcam), phospho-LimK (CST), cofilin and phospho-cofilin (CST), detyrosinated tubulin (glu-tubulin; Abcam), vimentin (Santa Cruz) HSP90 (CST), and GAPDH (Ambion). Densitometry was performed using ImageJ software.

### RhoGTPase Pulldown

MCF10 cells expressing shCtrl or shObsc-1 or -2 were grown as for attached or suspended western blots, collected and used for Rho GTPase pulldowns as per the manufacturer's protocols (Cytoskeleton, Inc). Densitometry was performed using ImageJ software.

### Confocal Microscopy

Cells were grown on glass coverslips and fixed with 4% paraformaldehyde as previously described [22]. For ROCK inhibition, cells were treated with 10 μM Y27632 in complete media for 1 hour prior to fixation. Cells were stained with phalloidin-568 (Molecular Probes), and representative regions were imaged on a Zeiss LSM510 confocal microscope under 60X magnification.

### Transfections

MCF10A cells expressing shObsc were seeded at 40% confluence and 24 hours later transfected with empty pmCherry-C1 vector (Clontech) or with pmCherry-C1 vector containing the human obscurin RhoGEF domain (amino acids 5697-5873, inclusive, of NCBI NP_443075). The TransIT-2020 reagent (Mirus) was used to achieve approximately 80% transfection efficiency, and cells used for blotting, RhoGTPase pulldowns, or McTN formation 24 hours post-transfection.

### Scratch Assay

shObsc-1 and -2 MCF10A cells transfected with empty vector or the obscurin RhoGEF domain were detached 24 hours post-transfection and plated overnight at 800,000 cells per well of a 12-well dish to achieve 100% confluence. A 200 μL pipet tip was used to introduce a scratch in the monolayer, and fresh complete media with or without 10 μM Y27632 was added to remove debris. Images were collected at 0 and 6 hours, and measurement of wound width was performed using ImageJ software, blindly.

### Invasion Assay

shObsc-1 and shObsc-2 MCF10A cells transfected with empty vector or the obscurin RhoGEF domain were detached 24 hours post-transfection, spun down, and resuspended in DMEM, then added to the interior of 24-well format Transwell chambers (Corning) at 150,000 cells per chamber. Complete MCF10A media was added to the exterior of the chamber and cells allowed to invade towards the chemoattractant. For cells treated with ROCK inhibitor, 10 μM Y27632 was included in both the inner and outer chambers. After 16 hours, the membrane surface was scrubbed to remove non-invading cells, and fixed with 0.5% crystal violet in 50% methanol. Invading cells were photographed and blindly counted per membrane.

### PARP Cleavage

shCtrl-, shObsc-1-, and shObsc-2-expressing MCF10A cells were plated in low-attach conditions in serum- and growth factor-free media and collected in RIPA buffer after 24 hours. Equal amounts of lysates were blotted as previously described [12], and probed for total PARP (CST), which detects both intact (112 kDa) and cleaved (85 kDa) forms. For ROCK inhibition, Y27632 was added to the serum- and growth factor-free media at 10 μM for 24 hours prior to collection.

### Microtentacle Formation

MCF10A cells expressing shCtrl or shObsc-1 or -2 were transfected with Cell Mask Orange (Life Technologies) as a contrast agent and detached after 30 minutes, as previously described [14, 15]. 25,000 cells were added to 24-well low-attach plates in DMEM/F12 with 1% charcoal dextran-stripped fetal bovine serum (HyClone). After 30 minutes in suspension, 100 cells (approximately 10 fields of view at 60x) from 2 replicate wells were blindly scored on a fluorescent microscope for the presence of microtentacles. Experiments were completed 3 times. Images were color inverted in ImageJ for ease of viewing.

### Impedance Assay

The xCelligence system (Roche) was used to measure electrical impedance during attachment. MCF10A cells were grown to 50% confluence, detached, and 10,000 cells/well were allowed to attach to the xCelligence plate for two hours in complete media. Impedance (ΔC) is expressed in arbitrary units. For experiments in the presence of paclitaxel, cells were pre-treated with 0.5 μg/mL paclitaxel or vehicle control for 1 hour, then trypsinized and allowed to attach for an additional 2 hours in the presence of 0.5 μg/mL paclitaxel or vehicle. Experiments were repeated at least three times, and error bars represent the standard deviations of three replicate wells per experiment.

### XTT Assay

shCtrl, shObsc-1 and shObsc-2 cells were plated in 96-well dishes and grown to 50% confluence, then treated with 0.1 – 1.0 μM paclitaxel or vehicle for 48 hours in complete media. An XTT assay was performed as previously described [22] to measure viability relative to vehicle control. Values were normalized to control shRNA.

### Statistics

All experiments were performed at least three times and statistical significance achieved at *p*<0.05 by t-test. Except where noted, error bars represent the standard deviation (S.D.) of three experiments.

## SUPPLEMENTARY MATERIAL FIGURES



## References

[1] Hanahan D, Weinberg RA (2000). The hallmarks of cancer. Cell.

[2] Hanahan D, Weinberg RA (2011). Hallmarks of cancer: the next generation. Cell.

[3] Martin GS (2003). Cell signaling and cancer. Cancer cell.

[4] Hall A (2009). The cytoskeleton and cancer. Cancer metastasis reviews.

[5] Sahai E (2005). Mechanisms of cancer cell invasion. Current opinion in genetics & development.

[6] Yamaguchi H, Condeelis J (2007). Regulation of the actin cytoskeleton in cancer cell migration and invasion. Biochimica et biophysica acta.

[7] Etienne-Manneville S, Hall A (2002). Rho GTPases in cell biology. Nature.

[8] Parri M, Chiarugi P (2010). Rac and Rho GTPases in cancer cell motility control. Cell communication and signaling : CCS.

[9] Sanz-Moreno V, Gadea G, Ahn J, Paterson H, Marra P, Pinner S, Sahai E, Marshall CJ (2008). Rac activation and inactivation control plasticity of tumor cell movement. Cell.

[10] Vega FM, Ridley AJ (2008). Rho GTPases in cancer cell biology. FEBS letters.

[11] Sahai E, Olson MF, Marshall CJ (2001). Cross-talk between Ras and Rho signalling pathways in transformation favours proliferation and increased motility. The EMBO journal.

[12] Vitolo MI, Boggs AE, Whipple RA, Yoon JR, Thompson K, Matrone MA, Cho EH, Balzer EM, Martin SS (2013). Loss of PTEN induces microtentacles through PI3K-independent activation of cofilin. Oncogene.

[13] Krueger EW, Orth JD, Cao H, McNiven MA (2003). A dynamin-cortactin-Arp2/3 complex mediates actin reorganization in growth factor-stimulated cells. Molecular biology of the cell.

[14] Whipple RA, Balzer EM, Cho EH, Matrone MA, Yoon JR, Martin SS (2008). Vimentin filaments support extension of tubulin-based microtentacles in detached breast tumor cells. Cancer research.

[15] Whipple RA, Cheung AM, Martin SS (2007). Detyrosinated microtubule protrusions in suspended mammary epithelial cells promote reattachment. Experimental cell research.

[16] Balzer EM, Whipple RA, Thompson K, Boggs AE, Slovic J, Cho EH, Matrone MA, Yoneda T, Mueller SC, Martin SS (2010). c-Src differentially regulates the functions of microtentacles and invadopodia. Oncogene.

[17] Matrone MA, Whipple RA, Thompson K, Cho EH, Vitolo MI, Balzer EM, Yoon JR, Ioffe OB, Tuttle KC, Tan M, Martin SS (2010). Metastatic breast tumors express increased tau, which promotes microtentacle formation and the reattachment of detached breast tumor cells. Oncogene.

[18] Balzer EM, Whipple RA, Cho EH, Matrone MA, Martin SS (2010). Antimitotic chemotherapeutics promote adhesive responses in detached and circulating tumor cells. Breast cancer research and treatment.

[19] Matrone MA, Whipple RA, Balzer EM, Martin SS (2010). Microtentacles tip the balance of cytoskeletal forces in circulating tumor cells. Cancer research.

[20] Balakrishnan A, Bleeker FE, Lamba S, Rodolfo M, Daniotti M, Scarpa A, van Tilborg AA, Leenstra S, Zanon C, Bardelli A (2007). Novel somatic and germline mutations in cancer candidate genes in glioblastoma, melanoma, and pancreatic carcinoma. Cancer research.

[21] Sjoblom T, Jones S, Wood LD, Parsons DW, Lin J, Barber TD, Mandelker D, Leary RJ, Ptak J, Silliman N, Szabo S, Buckhaults P, Farrell C, Meeh P, Markowitz SD, Willis J (2006). The consensus coding sequences of human breast and colorectal cancers. Science.

[22] Perry NA, Shriver M, Mameza MG, Grabias B, Balzer E, Kontrogianni-Konstantopoulos A (2012). Loss of giant obscurins promotes breast epithelial cell survival through apoptotic resistance. FASEB journal : official publication of the Federation of American Societies for Experimental Biology.

[23] Shriver M, Stroka K, Vitolo MI, Martin SS, Huso D, Konstantopoulos K, Kontrogianni-Konstantopoulos A (2014). Loss of Giant Obscurins from Breast Epithelium Disrupts Cell Adhesion, Induces Epithelial to Mesenchymal Transition and Enhances Cell Motility and Invasion. Oncogene.

[24] Borisov AB, Kontrogianni-Konstantopoulos A, Bloch RJ, Westfall MV, Russell MW (2004). Dynamics of obscurin localization during differentiation and remodeling of cardiac myocytes: obscurin as an integrator of myofibrillar structure. The journal of histochemistry and cytochemistry : official journal of the Histochemistry Society.

[25] Borisov AB, Martynova MG, Russell MW (2008). Early incorporation of obscurin into nascent sarcomeres: implication for myofibril assembly during cardiac myogenesis. Histochemistry and cell biology.

[26] Borisov AB, Raeker MO, Russell MW (2008). Developmental expression and differential cellular localization of obscurin and obscurin-associated kinase in cardiac muscle cells. Journal of cellular biochemistry.

[27] Kontrogianni-Konstantopoulos A, Bloch RJ (2005). Obscurin: a multitasking muscle giant. Journal of muscle research and cell motility.

[28] Kontrogianni-Konstantopoulos A, Catino DH, Strong JC, Randall WR, Bloch RJ (2004). Obscurin regulates the organization of myosin into A bands. American journal of physiology Cell physiology.

[29] Kontrogianni-Konstantopoulos A, Catino DH, Strong JC, Sutter S, Borisov AB, Pumplin DW, Russell MW, Bloch RJ (2006). Obscurin modulates the assembly and organization of sarcomeres and the sarcoplasmic reticulum. FASEB journal : official publication of the Federation of American Societies for Experimental Biology.

[30] Raeker MO, Russell MW (2011). Obscurin depletion impairs organization of skeletal muscle in developing zebrafish embryos. Journal of biomedicine & biotechnology.

[31] Russell MW, Raeker MO, Korytkowski KA, Sonneman KJ (2002). Identification, tissue expression and chromosomal localization of human Obscurin-MLCK, a member of the titin and Dbl families of myosin light chain kinases. Gene.

[32] Young P, Ehler E, Gautel M (2001). Obscurin, a giant sarcomeric Rho guanine nucleotide exchange factor protein involved in sarcomere assembly. The Journal of cell biology.

[33] Fukuzawa A, Idowu S, Gautel M (2005). Complete human gene structure of obscurin: implications for isoform generation by differential splicing. Journal of muscle research and cell motility.

[34] Hu LY, Kontrogianni-Konstantopoulos A (2013). The kinase domains of obscurin interact with intercellular adhesion proteins. FASEB journal : official publication of the Federation of American Societies for Experimental Biology.

[35] Ackermann MA, Shriver M, Perry NA, Hu LY, Kontrogianni-Konstantopoulos A (2014). Obscurins: Goliaths and Davids take over non-muscle tissues. PloS one.

[36] Ford-Speelman DL, Roche JA, Bowman AL, Bloch RJ (2009). The rho-guanine nucleotide exchange factor domain of obscurin activates rhoA signaling in skeletal muscle. Molecular biology of the cell.

[37] Chrzanowska-Wodnicka M, Burridge K (1996). Rho-stimulated contractility drives the formation of stress fibers and focal adhesions. The Journal of cell biology.

[38] Even-Ram S, Doyle AD, Conti MA, Matsumoto K, Adelstein RS, Yamada KM (2007). Myosin IIA regulates cell motility and actomyosin-microtubule crosstalk. Nature cell biology.

[39] Salhia B, Rutten F, Nakada M, Beaudry C, Berens M, Kwan A, Rutka JT (2005). Inhibition of Rho-kinase affects astrocytoma morphology, motility, and invasion through activation of Rac1. Cancer research.

[40] Darenfed H, Dayanandan B, Zhang T, Hsieh SH, Fournier AE, Mandato CA (2007). Molecular characterization of the effects of Y-27632. Cell motility and the cytoskeleton.

[41] Yang S, Kim HM (2014). ROCK inhibition activates MCF-7 cells. PloS one.

[42] Vishnubhotla R, Bharadwaj S, Sun S, Metlushko V, Glover SC (2012). Treatment with Y-27632, a ROCK Inhibitor, Increases the Proinvasive Nature of SW620 Cells on 3D Collagen Type 1 Matrix. International journal of cell biology.

[43] Frisch SM, Francis H (1994). Disruption of epithelial cell-matrix interactions induces apoptosis. The Journal of cell biology.

[44] Martin SS, Leder P (2001). Human MCF10A mammary epithelial cells undergo apoptosis following actin depolymerization that is independent of attachment and rescued by Bcl-2. Molecular and cellular biology.

[45] Frisch SM, Screaton RA (2001). Anoikis mechanisms. Current opinion in cell biology.

[46] Bharadwaj S, Thanawala R, Bon G, Falcioni R, Prasad GL (2005). Resensitization of breast cancer cells to anoikis by tropomyosin-1: role of Rho kinase-dependent cytoskeleton and adhesion. Oncogene.

[47] Watanabe K, Ueno M, Kamiya D, Nishiyama A, Matsumura M, Wataya T, Takahashi JB, Nishikawa S, Nishikawa S, Muguruma K, Sasai Y (2007). A ROCK inhibitor permits survival of dissociated human embryonic stem cells. Nature biotechnology.

[48] Zhang L, Valdez JM, Zhang B, Wei L, Chang J, Xin L (2011). ROCK inhibitor Y-27632 suppresses dissociation-induced apoptosis of murine prostate stem/progenitor cells and increases their cloning efficiency. PloS one.

[49] Liu X, Ory V, Chapman S, Yuan H, Albanese C, Kallakury B, Timofeeva OA, Nealon C, Dakic A, Simic V, Haddad BR, Rhim JS, Dritschilo A, Riegel A, McBride A, Schlegel R (2012). ROCK inhibitor and feeder cells induce the conditional reprogramming of epithelial cells. The American journal of pathology.

[50] Li X, Meng G, Krawetz R, Liu S, Rancourt DE (2008). The ROCK inhibitor Y-27632 enhances the survival rate of human embryonic stem cells following cryopreservation. Stem cells and development.

[51] Chapman S, Liu X, Meyers C, Schlegel R, McBride AA (2010). Human keratinocytes are efficiently immortalized by a Rho kinase inhibitor. The Journal of clinical investigation.

[52] Ohgushi M, Matsumura M, Eiraku M, Murakami K, Aramaki T, Nishiyama A, Muguruma K, Nakano T, Suga H, Ueno M, Ishizaki T, Suemori H, Narumiya S, Niwa H, Sasai Y (2010). Molecular pathway and cell state responsible for dissociation-induced apoptosis in human pluripotent stem cells. Cell stem cell.

[53] Camara O, Rengsberger M, Egbe A, Koch A, Gajda M, Hammer U, Jorke C, Rabenstein C, Untch M, Pachmann K (2007). The relevance of circulating epithelial tumor cells (CETC) for therapy monitoring during neoadjuvant (primary systemic) chemotherapy in breast cancer. Annals of oncology : official journal of the European Society for Medical Oncology / ESMO.

[54] Pantel K, Brakenhoff RH (2004). Dissecting the metastatic cascade. Nature reviews Cancer.

[55] (2012). The Cancer Genome Atlas Network Comprehensive molecular portraits of human breast tumours. Nature.

[56] Kontrogianni-Konstantopoulos A, Jones EM, Van Rossum DB, Bloch RJ (2003). Obscurin is a ligand for small ankyrin 1 in skeletal muscle. Molecular biology of the cell.

