# Supplementary material for: Loss of the obscurin-RhoGEF downregulates RhoA signaling and increases microtentacle formation and attachment of breast epithelial cells
Source: Oncotarget. 2014 Aug 10;5(18):8558–68. doi: 10.18632/oncotarget.2338 (PMC4226704; doi:10.18632/oncotarget.2338)
Supplement: Supplementary file 1 [file oncotarget-05-8558-s001.pdf]

Loss of the obscurin-RhoGEF downregulates RhoA signaling and increases microtentacle formation and attachment of breast epithelial cells

Supplementary Material

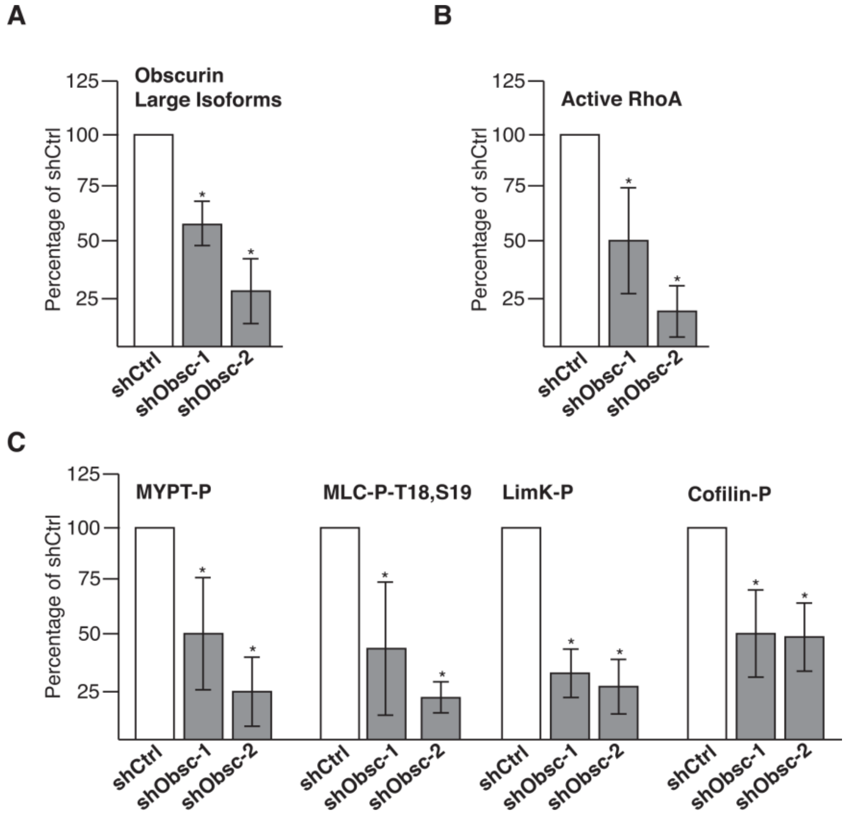

Supplementary Figure 1: Densitometry of attached cells. A) Western blots of obscurins were performed three times and densitometry of the largest isoforms, A and B, was quantified using ImageJ software. Bands were normalized to the loading control, tubulin. N=3. Error Bars: +/- S.D. Asterisks: $p<0.05$. B) Active RhoA pulldowns in attached cells were performed three times and densitometry measured with ImageJ software. Bands were normalized to total RhoA and the loading control, HSP90. N=3. Error Bars: +/- S.D. Asterisks: $p<0.05$. C) Western blots of attached cells were performed three times and densitometry of phospho-MYPT, -MLC, -LimK, and -cofilin measured with Image J software. Bands were normalized to the loading control, HSP90. N=3. Error Bars: +/- S.D. Asterisks: $p<0.05$.

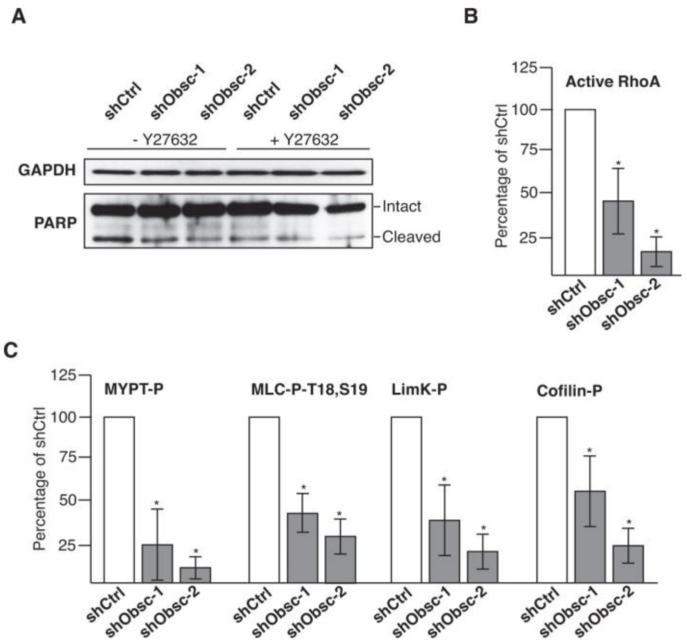

Supplementary Figure 2: Densitometry of suspended cells. A) Active RhoA pulldowns in suspended cells were performed three times and densitometry was measured with ImageJ software. Bands were normalized to total RhoA and the loading control, HSP90. N=3. Error Bars: +/- S.D. Asterisks: $p<0.05$. B) Western blots of suspended cells were performed three times and densitometry of phospho-MYPT, -MLC, -LimK, and -cofilin measured with Image J software. Bands were normalized to the loading control, HSP90. N=3. Error Bars: +/- S.D. Asterisks: $p<0.05$.

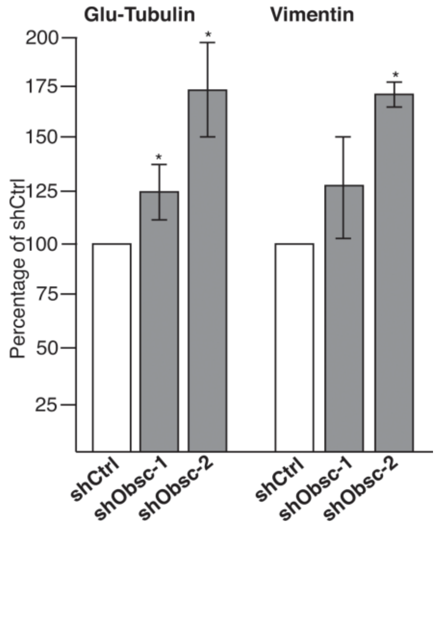

Supplementary Figure 3: Densitometry of glu-tubulin and vimentin. Western blots of glu-tubulin and vimentin were performed three times and densitometry was measured with ImageJ software. Bands were normalized to the loading control, GAPDH. N=3. Error Bars: +/- S.D.
